# Supplementary material for: Health Impact and Risk Factors Affecting South and Southeast Asian Women Following Natural Disasters: A Systematic Review
Source: Int J Environ Res Public Health. 2021 Oct 21;18(21):11068. doi: 10.3390/ijerph182111068 (PMC8611646; doi:10.3390/ijerph182111068)
Supplement: Supplementary file 1 [file ijerph-18-11068-s001.zip › Tables S2 and S3.pdf]

Table S2: Search terms and search strategy for each database

PubMed

MeSH terms used in this database. MeSH is the US National Library of Medicine (NLM)'s-controlled vocabulary or thesaurus of **terms** used to organise the MEDLINE database. It is also used for searching in PubMed, and some other databases, such as CINAHL, and the Cochrane Library.

Search date, search field, limits,	Search terms	Results
20 May, 2021<br><br>1st Searched in Title/Abstract	("Women"[Mesh] OR women OR woman OR female) AND ("Health"[Mesh] OR (health (vulnerability OR vulnerabilities OR risk OR risks OR hazard OR hazards OR psychological OR psychosocial OR mental OR physical OR emotion OR emotional OR reproductive OR sexual))) AND (("Natural Disasters"[Mesh] OR "natural disaster" OR "natural disasters" OR "natural calamity" OR natural calamities OR flood OR flooding OR volcano OR volcanic OR earthquake OR earthquakes OR cyclone OR cyclones OR hurricane OR drought OR tornado OR tornadoes OR landslide OR mudslide OR "wildfire" OR bushfire)) AND ("south Asia" OR "south-east Asia" OR bangladesh OR srilanka OR sri Lanka OR india OR bhutan OR nepal OR pakistan OR maldives OR afghanistan OR brunei OR burma OR cambodia OR indonesia OR laos OR malaysia OR phillippines OR singapore OR thailand OR vietnam)	457
20 May, 2021<br><br>Filters:<br>Journal Article, Humans, English, Female, from 2008/6/1 - 2021/3/31	((("Women"[Mesh] OR women OR woman OR gender OR female*) AND ("Health"[Mesh] OR (health (vulnerable* OR risk OR hazard* OR psychological OR mental OR physical OR emotion* OR sexual)))) AND (("Natural Disasters"[Mesh] OR "natural disaster*" OR "natural calamit*" OR flood* OR volcan* OR earthquake* OR cyclone* OR hurricane* OR drought* OR tornado* OR landslide* OR mudslide* OR "wild fire*" OR "wildfire*" OR bushfire* OR Vegetation Fires*) AND ("Natural Disasters"[Mesh] OR "natural disaster*" OR "natural calamit*" OR flood* OR volcan* OR earthquake* OR cyclone* OR hurricane* OR drought* OR tornado* OR landslide* OR mudslide* OR "wild fire*" OR "wildfire*" OR bushfire* OR Vegetation Fires*))) AND ("south Asia" OR "south-east Asia" OR Bangladesh OR Srilanka OR Sri Lanka OR India OR Bhutan OR Nepal OR Pakistan OR Maldives OR Afghanistan OR Brunei OR Burma OR Cambodia OR Indonesia OR Laos OR Malaysia OR Phillippines OR Singapore OR Thailand OR Vietnam) Filters: Full text, Journal Article, Humans, English, Female, from 2008/6/1 - 2021/3/31	358

ProQuest Database: Searched in 9 Databases

Search date, search field, limits,	Search terms	Results
20 May, 2021<br><br>1st Searched in Title OR Abstract OR Main subject	(women OR woman OR female OR females) AND (health (vulnerability OR vulnerabilities OR risk OR risks OR hazard OR hazards OR hazardous OR psychological OR mental OR physical OR emotion OR emotional OR psychosocial OR reproductive OR sexual)) AND (("natural disasters" OR "natural disaster") OR ("natural calamities" OR "natural calamity") OR flood OR floods OR flooding OR volcano OR volcanoes OR volcanic OR earthquake OR earthquakes OR cyclone OR cyclones OR hurricane OR hurricanes OR drought OR droughts OR tornado OR tornadoes OR landslide OR landslides OR mudslide OR mudslides OR ("wildfire" OR "wildfires") OR "wildfire" OR "wildfires" OR bushfire OR bushfires) AND ("south Asia" OR "south-east Asia" OR Bangladesh OR SriLanka OR Sri Lanka OR India OR Bhutan OR Nepal OR Pakistan OR Maldives OR Afghanistan OR Brunei OR Burma OR Myanmar OR Cambodia OR Timo-Leste OR Indonesia OR Laos OR Malaysia OR Philippines OR Singapore OR Thailand OR Vietnam)<br><br>OR<br><br>ti((women OR woman OR female OR females) AND (health (vulnerability OR vulnerabilities OR risk OR risks OR hazard OR hazards OR hazardous OR psychological OR mental OR physical OR emotion OR emotional OR psychosocial OR reproductive OR sexual)) AND (("natural disasters" OR "natural disaster") OR ("natural calamities" OR "natural calamity") OR flood OR floods OR flooding OR volcano OR volcanoes OR volcanic OR earthquake OR earthquakes OR cyclone OR cyclones OR hurricane OR hurricanes OR drought OR droughts OR tornado OR tornadoes OR landslide OR landslides OR mudslide OR mudslides OR ("wildfire" OR "wildfires") OR "wildfire" OR "wildfires" OR bushfire OR bushfires) AND ("south Asia" OR "south-east Asia" OR Bangladesh OR SriLanka OR Sri Lanka OR India OR Bhutan OR Nepal OR Pakistan OR Maldives OR Afghanistan OR Brunei OR Burma OR Myanmar OR Cambodia OR Timo-Leste OR Indonesia OR Laos OR Malaysia OR Philippines OR Singapore OR Thailand OR Vietnam)) OR ab((women OR woman OR female OR females) AND (health (vulnerability OR vulnerabilities OR risk OR risks OR hazard OR hazards OR hazardous OR psychological OR mental OR physical OR emotion OR emotional OR psychosocial OR reproductive OR sexual)) AND (("natural disasters" OR "natural disaster") OR ("natural calamities" OR "natural calamity") OR flood OR floods OR flooding OR volcano OR volcanoes OR volcanic OR earthquake OR earthquakes OR cyclone OR cyclones OR hurricane OR hurricanes OR drought OR droughts OR tornado OR tornadoes OR landslide OR landslides OR mudslide OR	528

	mudslides OR ("wildfire" OR "wildfires") OR "wildfire" OR "wildfires" OR bushfire OR bushfires) AND ("south Asia" OR "south-east Asia" OR Bangladesh OR SriLanka OR Sri Lanka OR India OR Bhutan OR Nepal OR Pakistan OR Maldives OR Afghanistan OR Brunei OR Burma OR Myanmar OR Cambodia OR Timo-Leste OR Indonesia OR Laos OR Malaysia OR Philippines OR Singapore OR Thailand OR Vietnam)) OR su((women OR woman OR female OR females) AND (health (vulnerability OR vulnerabilities OR risk OR risks OR hazard OR hazards OR hazardous OR psychological OR mental OR physical OR emotion OR emotional OR psychosocial OR reproductive OR sexual)) AND (("natural disasters" OR "natural disaster") OR ("natural calamities" OR "natural calamity") OR flood OR floods OR flooding OR volcano OR volcanoes OR volcanic OR earthquake OR earthquakes OR cyclone OR cyclones OR hurricane OR hurricanes OR drought OR droughts OR tornado OR tornadoes OR landslide OR landslides OR mudslide OR mudslides OR ("wildfire" OR "wildfires") OR "wildfire" OR "wildfires" OR bushfire OR bushfires) AND ("south Asia" OR "south-east Asia" OR Bangladesh OR SriLanka OR Sri Lanka OR India OR Bhutan OR Nepal OR Pakistan OR Maldives OR Afghanistan OR Brunei OR Burma OR Myanmar OR Cambodia OR Timo-Leste OR Indonesia OR Laos OR Malaysia OR Philippines OR Singapore OR Thailand OR Vietnam)) OR mainsubject((women OR woman OR female OR females) AND (health (vulnerability OR vulnerabilities OR risk OR risks OR hazard OR hazards OR hazardous OR psychological OR mental OR physical OR emotion OR emotional OR psychosocial OR reproductive OR sexual)) AND (("natural disasters" OR "natural disaster") OR ("natural calamities" OR "natural calamity") OR flood OR floods OR flooding OR volcano OR volcanoes OR volcanic OR earthquake OR earthquakes OR cyclone OR cyclones OR hurricane OR hurricanes OR drought OR droughts OR tornado OR tornadoes OR landslide OR landslides OR mudslide OR mudslides OR ("wildfire" OR "wildfires") OR "wildfire" OR "wildfires" OR bushfire OR bushfires) AND ("south Asia" OR "south-east Asia" OR Bangladesh OR SriLanka OR Sri Lanka OR India OR Bhutan OR Nepal OR Pakistan OR Maldives OR Afghanistan OR Brunei OR Burma OR Myanmar OR Cambodia OR Timo-Leste OR Indonesia OR Laos OR Malaysia OR Philippines OR Singapore OR Thailand OR Vietnam))	
20 May, 2021	Narrowed by:<br>Entered date: 2008-06-01 - 2021-03-31;<br>Source type: Scholarly Journals;<br>Language: English;<br>Peer reviewed: Peer reviewed	193

ProQuest Health and Medicine: 9 databases searched

Search date, search field, limits,	Search terms	Results
20 May, 2021<br><br>1st Searched in Title OR Abstract OR Main subject	ti((women OR woman OR female OR females) AND (health (vulnerability OR vulnerabilities OR risk OR risks OR hazard OR hazards OR hazardous OR psychological OR mental OR physical OR emotion OR emotional OR psycho-social OR reproductive OR sexual)) AND ((natural disasters OR natural disaster) OR (natural calamities OR natural calamity) OR flood OR floods OR flooding OR volcano OR volcanoes OR volcanic OR earthquake OR earthquakes OR cyclone OR cyclones OR hurricane OR hurricanes OR drought OR droughts OR tornado OR tornadoes OR landslide OR landslides OR mudslide OR mudslides OR (wildfire OR wildfires) OR wildfire OR wildfires OR bush fire OR bush fires) AND (South Asia OR south-east Asia OR Bangladesh OR SriLanka OR Sri Lanka OR India OR Bhutan OR Nepal OR Pakistan OR Maldives OR Afghanistan OR Brunei OR Burma OR Myanmar OR Cambodia OR Indonesia OR Laos OR Malaysia OR Philippines OR Singapore OR Thailand OR Vietnam)) OR ab((women OR woman OR female OR females) AND (health (vulnerability OR vulnerabilities OR risk OR risks OR hazard OR hazards OR hazardous OR psychological OR mental OR physical OR emotion OR emotional OR psycho-social OR reproductive OR sexual)) AND ((natural disasters OR natural disaster) OR (natural calamities OR natural calamity) OR flood OR floods OR flooding OR volcano OR volcanoes OR volcanic OR earthquake OR earthquakes OR cyclone OR cyclones OR hurricane OR hurricanes OR drought OR droughts OR tornado OR tornadoes OR landslide OR landslides OR mudslide OR mudslides OR (wildfire OR wildfires) OR wildfire OR wildfires OR bush fire OR bush fires) AND (South Asia OR south-east Asia OR Bangladesh OR SriLanka OR Sri Lanka OR India OR Bhutan OR Nepal OR Pakistan OR Maldives OR Afghanistan OR Brunei OR Burma OR Myanmar OR Cambodia OR Indonesia OR Laos OR Malaysia OR Philippines OR Singapore OR Thailand OR Vietnam)) OR mainsubject((women OR woman OR female OR females) AND (health (vulnerability OR vulnerabilities OR risk OR risks OR hazard OR hazards OR hazardous OR psychological OR mental OR physical OR emotion OR emotional OR psycho-social OR reproductive OR sexual)) AND ((natural disasters OR natural disaster) OR (natural calamities OR natural calamity) OR flood OR floods OR flooding OR volcano OR volcanoes OR volcanic OR earthquake OR earthquakes OR cyclone OR cyclones OR hurricane OR hurricanes OR drought OR droughts OR tornado OR tornadoes OR landslide OR landslides OR mudslide OR mudslides OR (wildfire OR wildfires) OR wildfire OR wildfires OR bush fire OR bush fires) AND (South Asia OR south-east Asia OR Bangladesh OR SriLanka OR Sri Lanka OR India OR Bhutan OR Nepal OR Pakistan OR Maldives OR Afghanistan OR Brunei OR Burma OR Myanmar OR Cambodia OR Indonesia OR Laos OR Malaysia OR Philippines OR Singapore OR Thailand OR Vietnam))	168

20 May, 2021	Narrowed by:<br>Entered date: 2008-06-01 - 2021-03-31;<br>Source type: Scholarly Journals;<br>Language: English;<br>Peer reviewed: Peer reviewed	107

EBSCO database

Search date, search field, limits,	Search terms	Results
20 May, 2021<br><br>1st Searched in Title OR Abstract OR Subject	TI ((women OR woman OR female OR females) AND (health (vulnerability OR vulnerabilities OR risk OR risks OR hazard OR hazards OR hazardous OR psychological OR mental OR physical OR emotion OR emotional OR psychosocial OR reproductive OR sexual)) AND (("natural disasters" OR "natural disaster") OR ("natural calamities" OR "natural calamity") OR flood OR floods OR flooding OR volcano OR volcanoes OR volcanic OR earthquake OR earthquakes OR cyclone OR cyclones OR hurricane OR hurricanes OR drought OR droughts OR tornado OR tornadoes OR landslide OR landslides OR mudslide OR mudslides OR ("wildfire" OR "wildfires") OR "wildfire" OR "wildfires" OR bushfire OR bushfires) AND ((South Asia OR south-east Asia OR Bangladesh OR SriLanka OR Sri Lanka OR India OR Bhutan OR Nepal OR Pakistan OR Maldives OR Afghanistan OR Brunei OR Burma OR Myanmar OR Cambodia OR Indonesia OR Laos OR Malaysia OR Philippines OR Singapore OR Thailand OR Vietnam)))) OR<br><br>AB ((women OR woman OR female OR females) AND (health (vulnerability OR vulnerabilities OR risk OR risks OR hazard OR hazards OR hazardous OR psychological OR mental OR physical OR emotion OR emotional OR psychosocial OR reproductive OR sexual)) AND (("natural disasters" OR "natural disaster") OR ("natural calamities" OR "natural calamity") OR flood OR floods OR flooding OR volcano OR volcanoes OR volcanic OR earthquake OR earthquakes OR cyclone OR cyclones OR hurricane OR hurricanes OR drought OR droughts OR tornado OR tornadoes OR landslide OR landslides OR mudslide OR mudslides OR ("wildfire" OR "wildfires") OR "wildfire" OR "wildfires" OR bushfire OR bushfires) AND ((South Asia OR south-east Asia OR Bangladesh OR SriLanka OR Sri Lanka OR India OR Bhutan OR	157

	Nepal OR Pakistan OR Maldives OR Afghanistan OR Brunei OR Burma OR Myanmar OR Cambodia OR Indonesia OR Laos OR Malaysia OR Philippines OR Singapore OR Thailand OR Vietnam)))) OR<br><br>SU ((women OR woman OR female OR females) AND (health (vulnerability OR vulnerabilities OR risk OR risks OR hazard OR hazards OR hazardous OR psychological OR mental OR physical OR emotion OR emotional OR psychosocial OR reproductive OR sexual)) AND (("natural disasters" OR "natural disaster") OR ("natural calamities" OR "natural calamity") OR flood OR floods OR flooding OR volcano OR volcanoes OR volcanic OR earthquake OR earthquakes OR cyclone OR cyclones OR hurricane OR hurricanes OR drought OR droughts OR tornado OR tornadoes OR landslide OR landslides OR mudslide OR mudslides OR ("wildfire" OR "wildfires") OR "wildfire" OR "wildfires" OR bushfire OR bushfires) AND ((South Asia OR south-east Asia OR Bangladesh OR SriLanka OR Sri Lanka OR India OR Bhutan OR Nepal OR Pakistan OR Maldives OR Afghanistan OR Brunei OR Burma OR Myanmar OR Cambodia OR Indonesia OR Laos OR Malaysia OR Philippines OR Singapore OR Thailand OR Vietnam))))	
20 May, 2021	Narrowed by:<br>Entered date: 2008-06-01 - 2021-03-31;<br>Source type: Scholarly Journals;<br>Language: English;<br>Peer reviewed: Peer reviewed	121

Table S3: Risk of bias assessment.

Methodological quality of the included quantitative (descriptive) studies using MMAT (yes=1, no=0)									
**Study**	**Clear research objectives**	**Data addressing research objectives**	**Relevancy of sampling strategy**	**Sample representativeness of target population**	**Appropriateness of measurement**	**Low risk of nonresponse bias**	**Appropriateness of analysis to answer research question**	**Total points**	**Ratings (6-7=high quality, 4-5= medium, >4= low quality)**
Powell et al. (2019)	1	1	1	1	1	1	1	7	**high quality**
Aurizki et al. (2020)	1	1	1	1	1	1	1	7	**high quality**
Adhikari et al. (2019)	1	1	1	1	1	1	1	7	**high quality**
Mamun et al. (2019)	1	1	0	1	1	1	1	6	**high quality**
Schwind et al. (2019)	1	1	1	1	1	1	1	7	**high quality**
Bimali et al. (2018)	1	1	1	1	1	0	1	6	**high quality**
Dahal et al. (2018)	1	1	1	1	1	1	1	7	**high quality**
Feder et al. (2012)	1	1	6	1	1	1	1	6	**high quality**
George et al. (2012)	1	1	1	1	1	1	1	7	**high quality**
Naeem et al. (2011)	1	1	0	1	1	1	1	6	**high quality**
Methodological quality of the included qualitative studies using MMAT (yes=1, no=0)									
**Study**	**Clear research objectives**	**Data addressing research objectives**	**Appropriate approach to answer research question**	**Adequate data collection methods to address research question**	**Findings properly derived from data/ proper data analysis**	**Interpretation of results sufficiently verified by data**	**Coherence between data sources, collection, analysis, and interpretation**	**Total points**	**Ratings (6-7=high quality, 4-5= medium, >4= low quality)**
Adhikar et al. (2018)	1	1	1	1	1	1	1	7	**High quality**
Methodological quality of the included Mixed Method studies using MMAT (yes=1, no=0)									

**Study**	**Clear research objectives**	**Data addressing research objectives**	**adequate rationale for using a mixed method**	different components of the study effectively integrated	integration of qualitative and quantitative components adequately interpreted	Divergences and inconsistencies between quantitative and qualitative results adequately addressed?	different components of the study adhere to the quality criteria of each tradition of the methods involved	**Total points**	**Ratings (6-7=high quality, 4-5= medium, >4= low quality)**
Suhail et al. (2009)	1	1	0	1	1	0	0	4	**Medium**
Methodological quality of the included quantitative (non-randomized) studies using MMAT (yes=1, no=0)									
**Study**	**Clear research objectives**	**Data addressing research objectives**	**Participants representativeness**	**Measurements appropriate**	**Complete outcome data**	**Confounders accounted in design and analysis**	**Conducting intended intervention**	**Total points**	**Ratings (6-7=high quality, 4-5= medium, >4= low quality)**
Mondastri et al. (2012)	1	1	1	1	1	1	1	6	**High quality**
Pyari, et al. (2012)	1	1	1	1	1	0	1	6	**High quality**
Wickrama et al. (2011)	1	1	1	1	1	1	1	7	**High quality**
Ahmad et al. (2010)	0	1	1	1	1	0	1	5	**Medium**
